# Systemic Therapy Type and Timing Effects on Radiation Necrosis Risk in HER2+ Breast Cancer Brain Metastases Patients Treated With Stereotactic Radiosurgery

**DOI:** 10.3389/fonc.2022.854364

**Published:** 2022-05-20

**Authors:** Christine Park, Evan D. Buckley, Amanda E. D. Van Swearingen, Will Giles, James E. Herndon, John P. Kirkpatrick, Carey K. Anders, Scott R. Floyd

**Affiliations:** ^1^ Department of Medicine, Duke University Medical Center, Durham, NC, United States; ^2^ Duke Cancer Institute Biostatistics, Duke University Medical Center, Durham, NC, United States; ^3^ Duke Center for Brain and Spine Metastasis, Duke Cancer Institute, Duke University Medical Center, Durham, NC, United States; ^4^ Department of Radiation Oncology, Duke University Medical Center, Durham, NC, United States; ^5^ Department of Biostatistics and Bioinformatics, Duke University Medical Center, Durham, NC, United States; ^6^ Department of Neurosurgery, Duke University Medical Center, Durham, NC, United States

**Keywords:** breast cancer, brain metastasis, stereotactic radiotherapy, systemic therapy, radiation necrosis

## Abstract

**Background:**

There is a concern that HER2-directed systemic therapies, when administered concurrently with stereotactic radiosurgery (SRS), may increase the risk of radiation necrosis (RN). This study explores the impact of timing and type of systemic therapies on the development of RN in patients treated with SRS for HER2+ breast cancer brain metastasis (BCBrM).

**Methods:**

This was a single-institution, retrospective study including patients >18 years of age with HER2+ BCBrM who received SRS between 2013 and 2018 and with at least 12-month post-SRS follow-up. Presence of RN was determined *via* imaging at one-year post-SRS, with confirmation by biopsy in some patients. Demographics, radiotherapy parameters, and timing (“during” defined as four weeks pre- to four weeks post-SRS) and type of systemic therapy (e.g., chemotherapy, HER2-directed) were evaluated.

**Results:**

Among 46 patients with HER2+ BCBrM who received SRS, 28 (60.9%) developed RN and 18 (39.1%) did not based on imaging criteria. Of the 11 patients who underwent biopsy, 10/10 (100%) who were diagnosed with RN on imaging were confirmed to be RN positive on biopsy and 1/1 (100%) who was not diagnosed with RN was confirmed to be RN negative on biopsy. Age (mean 53.3 vs 50.4 years, respectively), radiotherapy parameters (including total dose, fractionation, CTV and size target volume, all *p*>0.05), and receipt of any type of systemic therapy during SRS (60.7% vs 55.6%, *p*=0.97) did not differ between patients who did or did not develop RN. However, there was a trend for patients who developed RN to have received more than one agent of HER2-directed therapy independent of SRS timing compared to those who did not develop RN (75.0% vs 44.4%, *p*=0.08). Moreover, a significantly higher proportion of those who developed RN received more than one agent of HER2-directed therapy *during* SRS treatment compared to those who did not develop RN (35.7% vs 5.6%, *p*=0.047).

**Conclusions:**

Patients with HER2 BCBrM who receive multiple HER2-directed therapies during SRS for BCBrM may be at higher risk of RN. Collectively, these data suggest that, in the eight-week window around SRS administration, if HER2-directed therapy is medically necessary, it is preferable that patients receive a single agent.

## Introduction

Breast cancer is the most common malignancy diagnosed in women and the second leading cause of cancer-related mortality among women worldwide (1). Increased survival has been observed in breast cancer patients due to advances in early diagnosis/screening methods and improved treatments. However, long-term survival is complicated by increased prevalence of breast cancer brain metastasis (BCBrM), which is associated with poor prognosis and decreased quality of life (2). Specifically, breast cancer is the second most common primary origin of BrM, with 15-30% of patients estimated to develop BrM during the course of advanced disease (3, 4).

Human epidermal growth factor receptor 2-positive (HER2+) breast cancer is a subtype of breast cancer with a predilection for BrM (5). As many as 30% of patients with advanced, metastatic HER2+ breast cancer will develop BrM (6). Current standard of care options for HER2+ BCBrM include radiation therapy (stereotactic radiosurgery [SRS] or whole brain radiation therapy [WBRT]), brain permeable systemic therapies, and/or neurosurgical resection when appropriate (7). A multimodal approach combining these different treatment modalities has improved the overall survival and functional outcomes of patients with BCBrM. Specifically for radiation therapy, SRS is a highly effective form of radiation therapy that offers meaningful control of BrM (8). Because the vast majority of patients who present with BrM have both intracranial and extracranial disease, most of them will also receive systemic treatment.

Radiation-induced injury is one of the most significant complications of brain tumor irradiation (9). One of the important adverse effects associated with SRS is radiation necrosis (RN), which is a late complication of radiation injury and occurs in about 5-25% of treated patients (10, 11). RN often significantly impacts quality of life; for example, it often presents with neurological deficits such as headaches, nausea and seizures (12, 13). The mechanism of RN remains unclear, but the pathology involves inflammation and angiogenesis in a region of coagulative necrosis associated with breakdown of the blood-brain barrier. resulting in perilesional edema and heterogeneous contrast enhancement (14, 15). RN commonly occurs 3-12 months after radiotherapy (16, 17), though it can be observed as late as several years post radiosurgery, in our experience. Because the likelihood of RN depends on factors such as timing of radiation therapy, total dose, dose per fraction and volume irradiated (18–20), efforts to decrease the rate of RN have focused on controlling these radiotherapy parameters. However, recent studies have shown that rates of RN are higher in patients who received both SRS and immunotherapies or targeted therapies compared to those who received SRS alone (21, 22). This association is significant because most BCBrM patients receive concurrent systemic therapy as part of their treatment regimen. This study explores the impact of timing and type of systemic therapies on the development of RN in patients with HER2+ BCBrM treated with SRS.

## Methods

This was a single-institution, retrospective study (approved by the Institutional Review Board) of patients >18 years of age with HER2+ BCBrM who received SRS between 2013 and 2018 with at least 12-month post-SRS follow-up. Demographics and baseline characteristics including age at the time of SRS, race, location of irradiated BrM, and number of BrM were collected. Relevant systemic and radiation treatment details were also recorded. Presence of RN was determined *via* magnetic resonance (MR) imaging one-year post-SRS (see details below). The rate of RN was also determined using biopsy reports of brain lesions if they were available at any time after SRS (i.e., not restricted to within one-year post-SRS). Patients with incomplete follow-up data and/or who were deceased within one-year post-SRS were excluded. Demographics and lesion characteristics considered included age at the time of SRS, race/ethnicity, time to SRS from date of brain metastasis, and location of brain metastasis.

### Brain Metastases

Location of irradiated BrM was categorized as follows: frontal, parietal, temporal, occipital, cerebellar, midbrain/brainstem, and multiple. The number of BrM was considered as a binary variable (single or multiple).

### Imaging and Pathological Criteria for Radiation Necrosis

MR imaging was used to diagnose RN within 12 months of receipt of SRS. For a given area, the diagnosis of RN was determined by: 1) the degree of hyperintensity on T2-weighted image and enhancement on contrast-enhanced T1-weighted image and 2) assessment and confirmation by neuroradiologists. For biopsy-confirmed cases with associated pathology reports, pure (tumor absent) and mixed (tumor present) RN were all considered as RN.

### Radiotherapy Parameters

The total administered dose (in grays) and the number of fractions were collected. Other relevant radiotherapy parameters considered were clinical target volume (CTV), gross tumor volume (GTV), conformity index (CI), and volume receiving 12 gray (V12Gy). For V12Gy, only single-fraction SRS was considered.

### Systemic Therapy

Types of systemic therapy included HER2-directed therapy (T-DM1, trastuzumab, pertuzumab, lapatinib), mitosis inhibitors (taxanes, vinca alkaloids, eribulin), DNA synthesis inhibitors (capecitabine, platinum, anthracycline, pemetrexed, cyclophosphamide, doxorubicin, gemcitabine), and other (all other systemic treatments). Timing of systemic therapy with regard to radiotherapy was defined as a binary variable as follows: 1) systemic therapy “during” radiotherapy meant the systemic therapy was administered within 4 weeks prior to Day 1 of SRS treatment through 4 weeks post-SRS Day 1; 2) “not during” radiotherapy meant systemic therapy was administered outside the 8-week window surrounding SRS. Number of systemic therapy agents overall, number of HER2-directed therapies, and use of T-DM1 were also considered.

### Statistical Analysis

Categorical variables were summarized with frequencies and percentages and analyzed using Pearson’s Chi-squared test with Yates’ continuity correction. Continuous variables were summarized with means/standard deviations and medians/minimum and maximum and analyzed using the Wilcoxon rank sum test with continuity correction. Statistical significance was assessed at level alpha = 0.05. All statistical analyses were conducted using both SAS 9.4 (SAS Institute Inc., Cary, NC, USA) and R (RStudio, Inc; Boston, MA).

## Results

**Figure 1:**
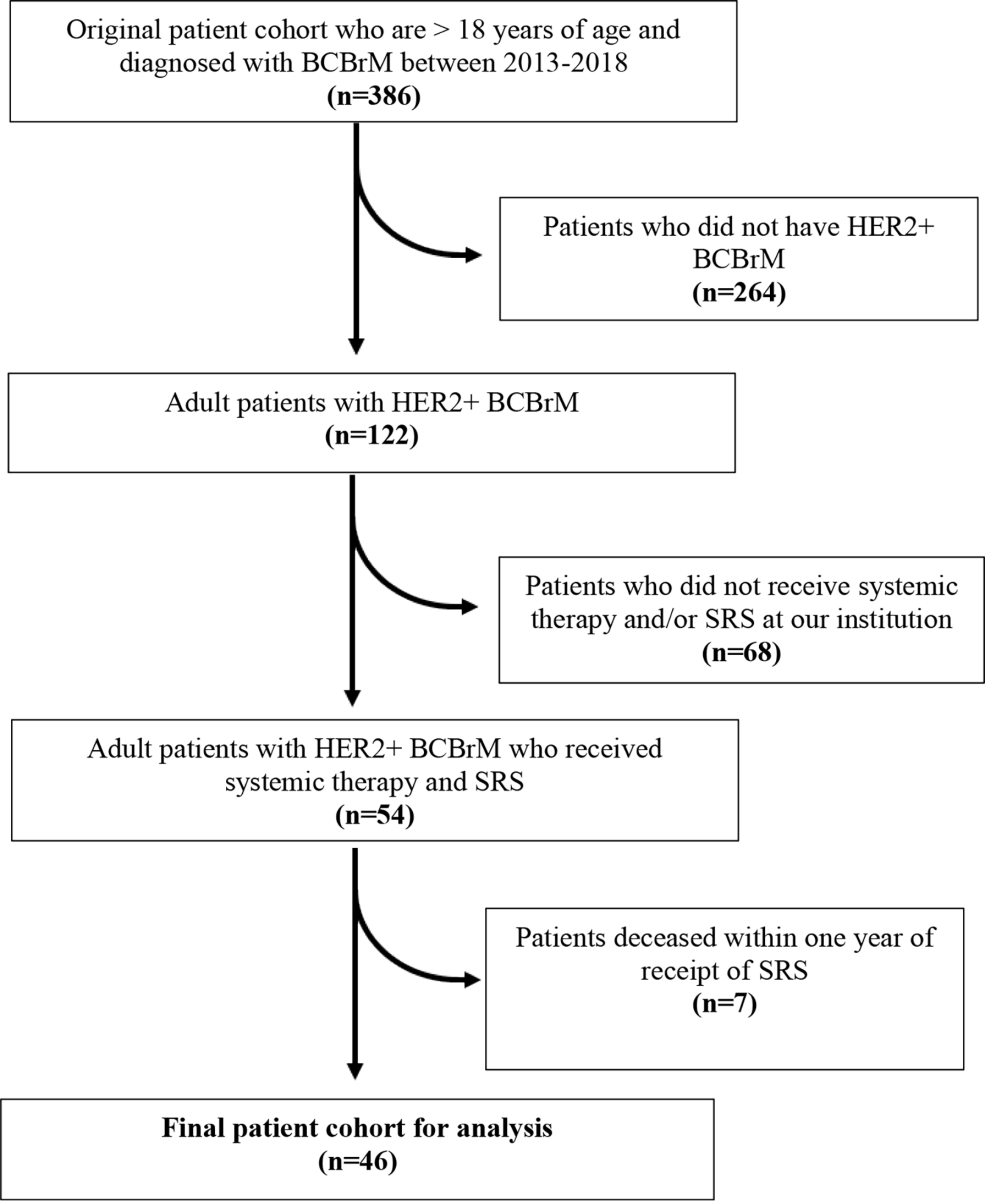
Consort diagram of selection of patient cohort. Inclusion and exclusion criteria applied for derivation of final sample cohort.

An initial sample of 386 adult patients who were diagnosed with BCBrM were identified between 2013 and 2018. From this sample, 264 patients were excluded as they did not have HER2+ BCBrM. 68 patients were further excluded as they did not receive systemic therapy and/or SRS at our institution. Finally, seven patients who were deceased within one year following SRS were excluded. A final cohort of 46 patients remained after applying these exclusion criteria (**Figure 1**).

### Patient Demographics and Lesion Characteristics

Among 46 patients with HER2+ BCBrM who received both SRS and systemic therapies, the mean age at time of SRS was 52.1 years and the cohort was predominantly white (76.1% vs 23.9% non-white). A majority of patients had a single treated BrM (63%) vs multiple (37%).

In this cohort of 46 patients, 28 (60.9%) developed RN and 18 (39.1%) did not based on imaging parameters. Of the 11 patients from whom tissue biopsy samples were obtained (average date of biopsy was 1.5 years after SRS), 10/10 (100%) patients who were diagnosed with RN on imaging were confirmed to have RN on biopsy (with 4 of those reported as pure RN and 6 as mixed RN/recurrence) and 1/1 (100%) patient who was determined to not have RN on imaging was confirmed to not have RN on biopsy. Age at time of SRS did not differ between those who developed RN and those who did not (mean 53.3 vs 50.4 years, respectively; *p*=0.54). There was a higher, though not statistically significant, percentage of African Americans in the RN group (28.6% vs 11.1%, *p*=0.24). Although there was no statistically significant difference between the anatomic location of BrM irradiation between the two cohorts, more patients who developed RN had a single BrM lesion (78.6%) as opposed to multiple BrM lesions (21.4%) (p=0.016). Conversely, more patients who did not develop RN had multiple BrM (61.1%) compared to those with a single lesion (38.9%) (*p*=0.016). The results are summarized in **Table 1**.

**Table 1:** Demographics and lesion characteristics.

Variable	No RN (n=18)	RN (n=28)	Total (n=46)	p-value
Age at time of SRS, years				0.54
Mean (SD)	50.4 (13.0)	53.3 (11.7)	52.1 (12.1)	
Median [Min, Max]	51.5 [28.0, 74.0]	55.0 [33.0, 78.0]	53.0 [28.0, 78.0]	
Race, n (%)				0.24
White	16 (88.9)	19 (67.9)	35 (76.1)	
Black	2 (11.1)	8 (28.6)	10 (21.7)	
Other	0 (0)	1 (3.6)	1 (2.2)	
Location of irradiated brain metastasis, n (%)				0.14
Frontal	3 (16.7)	4 (14.3)	12 (26.1)	
Parietal	1 (5.6)	4 (14.3)	5 (10.9)	
Temporal	0 (0)	2 (7.1)	2 (4.3)	
Occipital	0 (0)	2 (7.1)	2 (4.3)	
Cerebellar	3 (16.7)	9 (32.1)	12 (26.1)	
Midbrain/Brainstem	0 (0)	1 (3.6)	1 (2.2)	
Multiple	11 (61.1)	6 (21.4)	17 (37.0)	
Number of brain metastasis (binary), n (%)				**0.016**
Single	7 (38.9)	22 (78.6)	29 (63.0)	
Multiple	11 (61.1)	6 (21.4)	17 (37.0)	

RN, radiation necrosis; SD, standard deviation; SRS, stereotactic radiosurgery. Significant p values (p<0.05) in bold.

### Radiation Treatment

Overall, the mean total dose of SRS administered was 21.9 ± 4.10 Gy. 60.9% of the patients underwent single-fraction SRS. The mean values for the measured radiotherapy parameters were as follows: CTV of 9.15 ± 13.0 cc, GTV of 5.39 ± 7.51 cc, CI of 1.32 ± 0.27, and V12Gy (for single-fraction only) of 7.14 ± 6.28 cc. When we compared the two groups, there were no significant differences in the total dose, fraction (1 vs 5), and all measured radiotherapy parameters (all *p*>0.05) as shown in **Table 2**.

**Table 2:** Radiation parameters.

Variable	No RN (n=18)	RN (n=28)	Total (n=46)	p-value
Total dose (Gy)				0.19
Mean (SD)	22.9 (3.52)	21.3 (4.37)	21.9 (4.10)	
Median [Min, Max]	22.5 [18.0, 30.0]	20.0 [10.0, 27.5]	20.0 [10.0, 30.0]	
Fractions				>0.95
1	11 (61.1%)	17 (60.7%)	28 (60.9%)	
5	7 (38.9%)	11 (39.3%)	18 (39.1%)	
CTV (cc)				0.86
Mean (SD)	8.21 (11.3)	9.75 (14.2)	9.15 (13.0)	
Median [Min, Max]	5.74 [0.312, 44.9]	4.19 [0.104, 54.1]	5.15 [0.104, 54.1]	
Missing	2 (11.1%)	3 (10.7%)	5 (10.9%)	
GTV (cc) (single-fraction only)				0.84
Mean (SD)	4.72 (5.81)	5.82 (8.51)	5.39 (7.51)	
Median [Min, Max]	3.27 [0.138, 21.5]	2.60 [0.0264, 34.8]	2.63 [0.0264, 34.8]	
Missing	2 (18.2%)	2 (11.8%)	4 (14.3%)	
CI				0.55
Mean (SD)	1.30 (0.240)	1.34 (0.29)	1.32 (0.27)	
Median [Min, Max]	1.23 [1.03, 1.84]	1.29 [1.03, 2.35]	1.25 [1.03, 2.35]	
Missing	2 (11.1%)	3 (10.7%)	5 (10.9%)	
V12Gy (cc)				0.59
Mean (SD)	6.55 (6.74)	7.49 (6.20)	7.14 (6.28)	
Median [Min, Max]	3.96 [0.947, 20.2]	5.23 [1.66, 23.1]	5.18 [0.947, 23.1]	
Missing	2 (11.1%)	3 (10.7%)	5 (10.9%)	

CI, conformity index; CTV, clinical target volume; GTV, gross tumor volume; Gy, gray; SD, standard deviation; V12Gy, volume receiving 12Gy.

### Systemic Treatment

In the entire cohort, 58.7% of patients received any type of systemic therapy (i.e., HER2-directed therapy, mitosis inhibitors, DNA synthesis inhibitors, others) during SRS. Specifically, 43.5% received a HER2-directed therapy, 2.2% received a mitosis inhibitor therapy, 8.7% received both HER2-directed and mitosis inhibitor therapy, and 4.3% received other systemic therapy.

Receipt of any systemic therapy during SRS did not differ between patients who did or did not develop RN (60.7% vs 55.6%, *p*=0.97) (**Table 3**). However, patients who developed RN more commonly received more than one agent of HER2-directed therapy, independent of SRS timing, compared to those who did not develop RN (75.0% vs 44.4%, *p*=0.08). A significantly higher proportion of those who developed RN received more than one agent of HER2-directed therapy *during* SRS compared to those did not develop RN (35.7% vs 5.6%, *p*=0.047).

**Table 3:** Systemic therapy and SRS.

Variable	No RN (n=18)	RN (n=28)	Total (n=46)	p-value
Time to first SRS from date of BrM (mo)				0.84
Mean (SD)	4.65 (6.63)	4.33 (7.64)	4.46 (7.19)	
Median [Min, Max]	1.03 [0.131, 22.0]	0.986 [0, 28.5]	1.00 [0, 28.5]	
Agents of systemic therapies, n (%)				>0.95
≤4	11 (61.1%)	18 (64.3%)	29 (63.0%)	
>4	7 (38.9%)	10 (35.7%)	17 (37.0%)	
Systemic therapy during SRS, n (%)				0.97
No	8 (44.4%)	11 (39.3%)	19 (41.3%)	
Yes	10 (55.6%)	17 (60.7%)	27 (58.7%)	
Type of systemic therapy received during SRS, n (%)				0.62
No systemic therapy	8 (44.4%)	11 (39.3%)	19 (41.3%)	
HER2-directed inhibitors	6 (33.3%)	14 (50.0%)	20 (43.5%)	
Mitosis inhibitors	1 (5.6%)	0 (0%)	1 (2.2%)	
HER2-directed inhibitors + mitosis inhibitors	2 (11.1%)	2 (7.1%)	4 (8.7%)	
Other	1 (5.6%)	1 (3.6%)	2 (4.3%)	
HER2-directed inhibitor during SRS, n (%)				0.59
No	10 (55.6%)	12 (42.9%)	22 (47.8%)	
Yes	8 (44.4%)	16 (57.1%)	24 (52.2%)	
Number of HER2-directed inhibiting agents during SRS, n (%)				**0.047**
0-1	17 (94.4%)	18 (64.3%)	35 (76.1%)	
2	1 (5.6%)	10 (35.7%)	11 (23.9%)	

SRS, stereotactic radiosurgery; SD, standard deviation; T-DM1, trastuzumab emtansine. “During” SRS defined as 4 weeks prior to through 4 weeks after Day 1 of SRS (an 8 week window). Significant p values (p<0.05) in bold.

## Discussion

In our cohort of 46 patients with HER2+ breast cancer and BrM who received SRS, 60.9% of them were determined to have RN on imaging. Of the 11 patients with RN who had a BrM biopsy specimen to evaluate, 10/10 who were diagnosed with RN on imaging were positive for RN on biopsy and 1/1 (100%) who was not diagnosed with RN on imaging was negative for RN. While the radiation treatment parameters did not differ significantly between those who did and did not develop RN, patients with RN were more often treated with more than one agent of HER2-directed therapy during SRS, as defined by 4 weeks prior to or after radiosurgery.

### Challenges in Diagnosing Radiation Necrosis

There is no single best imaging modality used to diagnose RN. However, in current practice, MR imaging is the most common modality used to explore RN. Because it is often difficult to distinguish between RN and recurrence, surgical resection remains the best way to establish the histopathological diagnosis of RN along with providing relief from any symptomatic effects. Depending on the method of assessing RN, the rate of RN can vary widely from 7% (biopsy-proven) to 24% (imaging based) (20, 23). Hence, it is possible that our high rate of RN (60.9%) observed in our study could have been overestimated based on our primarily imaging-based diagnosis of RN. Biopsy was available for 10 of the 28 of those diagnosed with RN, of which all 10 were confirmed. If the rate of RN was calculated from biopsy-proven RN only, the rate would have been 21.7% which is still higher than those reported for biopsy-proven series (24, 25).

Although our ability to make a definitive diagnosis of RN versus recurrent tumor is currently based on histopathology, the pathology results can be limited by the variability of surgical site sampling which can give confounding results in the presence of both RN and tumor. To augment the current diagnostic capacity, attention has been focused on identifying the features and techniques that are important for making the distinction between RN and recurrence. For example, various imaging modalities using different sequences have been studied. Magnetic resonance spectroscopy, diffusion-weighted imaging, diffusion tensor imaging, and positron emission tomography (PET), among others, have surfaced as promising candidates for improving the current diagnostic rates (26, 27). Specifically for PET, fluciclovine is an amino acid radiotracer that has recently demonstrated good initial results for distinguishing RN from recurrent tumor among patients with brain metastases who were treated with SRS (28, 29). Furthermore, there also have been efforts to use radiomics and data mining to develop models that can effectively differentiate tumor from RN (30).

### Significance of the Use of SRS and HER2-Directed Therapy on RN

BCBrM remains a significant challenge in the current era of improved extracranial disease control, owing to the lower efficacy of many systemic therapies in the brain (31). Given that HER2-driven cancers seem to preferentially metastasize to the brain, and the apparent brain-penetrance of some therapies (32, 33), we focused on the HER2-directed therapies. The effect of combination of HER2- directed therapies and SRS on overall survival/local control and RN is still unclear. For example, numerous studies have illustrated the benefit of concurrent use of lapatinib and SRS on overall survival and RN. Parsai et al. demonstrated that the use of lapatinib at any time of SRS administration was associated with improved overall survival (27.3 vs 19.5 months, *p*=0.03) with a lower risk of RN (1.3% vs 6.3% at 12 months, *p*<0.01) compared to those who received SRS without lapatinib in patients with HER2+ BCBrM (34). Miller et al. also showed that concurrent use of lapatinib/HER2-directed antibody treatment with SRS was associated with a lower 12-month cumulative incidence of RN (1.3% vs. 6.3%, p=0.001) compared to those who only received SRS. Furthermore, Kim et al. reported that the use of concurrent lapatinib with SRS did not increase the risk of RN compared to those who underwent SRS only (1.0% vs 3.5%, *p*=0.13) (35). On the other hand, the outcomes for T-DM1 are suboptimal. Carlson et al. showed in their case series that the overall rate of clinically significant RN among the patients in the treatment group who received SRS and T-DM1 was 57% (36). Geraud et al. also reported that RN was observed in 50% of patients who received T-DM1 concurrently with SRS compared to those who received T-DM1 sequentially to SRS (37). Thus, prior studies suggest that different agents (i.e. lapatinib as a tyrosine kinase inhibitor vs T-DM1 as an antibody drug conjugate) within the same class (HER2-directed) may have different risks on development of RN. While the current study was unable to assess differences between specific agents due to low numbers, this is a question warranting further exploration in future larger studies.

Although previous studies have looked into the effect of a specific therapy on the rate of RN for patients undergoing SRS, they have not considered the effect of different types or the number of systemic therapies (particularly HER2-directed therapies) on the development of RN during/following SRS. We found that a higher number (2 or more) of HER2-directed agents administered during SRS may increase the risk of development of RN. This result can be interpreted in two ways: 1) the toxicity of the HER2-directed therapies increases when used in combination and concurrently with SRS or 2) use of more HER2-directed therapies leads to longer survival and the observed higher rate of RN is a result of time bias. That is, there is an increased risk for RN in patients who live longer after their SRS treatment, allowing more time to observe the natural progression of RN (38). This is further supported by the observation that the diagnosis of RN was not made in the patients who deceased before their one-year follow-up. Nevertheless, our results suggest that timing and number of HER2-directed therapies matter when considering SRS for this patient population. These findings may be used to identify the group of patients with HER2+ BCBrMs with the highest risk for RN who would be candidates for preventative strategies in the future.

### Limitations

Our study is limited by its retrospective design which is subject to selection and misclassification bias. Some patients with HER2+ BCBrM who received SRS and systemic therapy might not have been included in our cohort if they received their treatment outside of our institution and were screened out in the initial phase. Also, the seven patients who were diagnosed with RN *via* imaging only could have been misclassified because the difference between RN and local recurrence is difficult to discern with conventional imaging techniques. There could also be confounding factors that may be present that were not accounted for which could have affected the statistical significance of the results. Furthermore, our cohort is largely homogenous with regards to race which is predominantly Caucasian. Hence, the results may not be applicable to the greater population. Finally, the small sample size and diversity of treatment histories (e.g., the wide variety of systemic agents) contributed to the lack of power to detect statistical difference in the measured outcomes. The lack of adequate sample size also made it difficult to draw meaningful conclusions through logistic regression modeling.

## Conclusion

Patients with HER2+ BCBrM who receive multiple agents of HER2-directed therapy during SRS for BCBrM may be at higher risk of RN. These data suggest during the eight-week window around SRS administration, if HER2-directed therapy is medically necessary, use of a single HER2-directed agent may lead to lower RN rates. Further investigation of next generation HER2-directed therapies, particularly comparing specific agents, in a larger cohort of patients will help refine best practices to minimize RN.

## Data Availability Statement

The original contributions presented in the study are included in the article/supplementary material. Further inquiries can be directed to the corresponding author.

## Ethics Statement

The studies involving human participants were reviewed and approved by Duke University Institutional Review Board (IRB). Written informed consent for participation was not required for this study in accordance with the national legislation and the institutional requirements.

## Author Contributions

CP, AV, JK, SF, and CA were involved with the conception of the study. CP, EB, WG, and JH participated in analysis and interpretation of data. CP formulated the initial draft of the manuscript. All co-authors reviewed the final draft of the manuscript.

## Funding

This study was funded by the Translating Duke Health Initiative as Carey K. Anders, MD is a Duke Health Scholar.

## Conflict of Interest

JK reports research funding from Varian Medical Systems and the BioMimetix SBIR, consultant fees from Monteris and ownership of ClearSight RT, LLC. CA reports the following: Research funding: PUMA, Lilly, Merck, Seattle Genetics, Nektar, Tesaro, G1-Therapeutics, ZION, Novartis, Pfizer; Compensated consultant role: Genentech, Eisai, IPSEN, Seattle Genetics; Astra Zeneca, Novartis, Immunomedics, Elucida, Athenex; Royalties: UpToDate, Jones and Bartlet. SF reports the following: Salary support for unrelated research from: NIH R01 #NS1000866-04, American Cancer Society Research Scholar Grant #133394-RSG-19-030-01-DMC, NIH R38 #CA245204-02, and BioMimetix/NIH R44 #CA228694-02.

The remaining authors declare that the research was conducted in the absence of any commercial or financial relationships that could be construed as a potential conflict of interest.

## Publisher’s Note

All claims expressed in this article are solely those of the authors and do not necessarily represent those of their affiliated organizations, or those of the publisher, the editors and the reviewers. Any product that may be evaluated in this article, or claim that may be made by its manufacturer, is not guaranteed or endorsed by the publisher.
